# Supplementary material for: ATP Analogues for Structural Investigations: Case Studies of a DnaB Helicase and an ABC Transporter
Source: Molecules. 2020 Nov 12;25(22):5268. doi: 10.3390/molecules25225268 (PMC7698047; doi:10.3390/molecules25225268)
Supplement: Supplementary file 1 [file molecules-25-05268-s001.pdf]

Supplementary Materials

ATP Analogues for Structural Investigations: Case Studies of a DnaB Helicase and an ABC Transporter

Denis Lacabanne [1,2,†], Thomas Wiegand [1,*,†], Nino Wili [1], Maria I. Kozlova [3], Riccardo Cadalbert [1], Daniel Klose [1], Armen Y. Mulkidjanian [3,4], Beat H. Meier [1,*] and Anja Böckmann [5,*]

1 Laboratory of Physical Chemistry, ETH Zurich, 8093 Zurich, Switzerland; denis.lacabanne@mrc-mbu.cam.ac.uk (D.L.); nino.wili@phys.chem.ethz.ch (N.W.); Riccardo.Cadalbert@nmr.phys.chem.ethz.ch (R.C.); daniel.klose@phys.chem.ethz.ch (D.K.)
2 Medical Research Council Mitochondrial Biology Unit University of Cambridge, Cambridge Biomedical Campus, Keith Peters Building, Hills Road, Cambridge CB2 0XY, U.K.
3 Department of Physics, Osnabrueck University, 49069 Osnabrueck, Germany; makozlova@uni-osnabrueck.de (M.I.K.); armen.mulkidjanian@uni-osnabrueck.de (A.Y.M.)
4 School of Bioengineering and Bioinformatics and Belozersky Institute of Physico-Chemical Biology, Lomonosov Moscow State University, 119234 Moscow, Russia
5 Molecular Microbiology and Structural Biochemistry UMR 5086 CNRS/Université de Lyon, Labex Ecofect, 69367 Lyon, France
* Correspondence: thomas.wiegand@phys.chem.ethz.ch (T.W.); beme@ethz.ch (B.H.M.); bockmann@ibcp.fr (A.B.)
† These authors contributed equally to this work.
Academic editor: Marilisa Leone

Materials and Methods

Proteins productions

The production, purification and reconstitution of BmrA[89, 133] and DnaB[38, 82] were done as described in the literature. The proteins were produced in M9-medium with ^{13}C-glucose (2 g.L^{-1}) and ^{15}N-ammonium chloride (2 g.L^{-1}) as sole sources of carbon-13 and nitrogen-15. A first pre-culture of 50 mL (incubated at 37 °C 150 rpm) was used to inoculate a second pre-culture of 150 mL (incubated at 25 °C 150 rpm). A 2 L flask containing 850 mL of M9-medium was inoculated with the 150-mL pre-culture and incubated at 25 °C, 150 rpm. Induction was performed with 0.7 mM IPTG at OD_{600nm} 0.65 until the stationary phase was reached. The bacteria were harvested by a centrifugation step at 6,000 × *g* during 15 min and lysed by high pressure using a Microfluidics Microfluidizer® in 50 mM Tris-HCl pH 8.0, 5 mM $MgCl_2$, 1 mM DTT, benzonase, and EDTA-free protease inhibitor cocktail. The solution was centrifuged at 15,000 × *g* during 45 min.

For BmrA the 15,000 × *g* centrifuge step is followed by a second centrifugation step, at 200,000 × *g* during one hour, of the supernatant. The sedimented membranes were suspended in 50 mM

Tris-HCl pH 8.0, 1 mM EDTA, and 300 mM sucrose. The protein-containing membranes were diluted at 2 g.L^{-1} and solubilized using 1% DDM (m/v) then centrifuged at 100,000 × *g* for 1 h. The supernatant was loaded onto a Ni-NTA column (Qiagen) previously equilibrated with 50 mM Tris-HCl pH 8.0, 100 mM NaCl, 15% glycerol (v/v), 10 mM imidazole, and 0.2% DDM (m/v). The Ni-NTA column was washed with 50 mM Tris-HCl pH 8.0, 0.2% DDM (m/v) with containing 0.5 M NaCl, then 30 mM imidazole, 40 mM imidazole and the protein is eluted with 250 mM imidazole. The eluted protein was desalted using PD10 columns (PD10 - GE Healthcare Life Sciences) which were equilibrated with 50 mM Tris-HCl pH 8.0, 100 mM NaCl, 10% glycerol (v/v), and 0.2% DDM (m/v) and diluted four times with 50 mM Tris-HCl pH 8.0, 100 mM NaCl, and 5% glycerol. The solution was mixed with a homemade preparation of *B. subtilis* lipids solubilized in Triton X-100 with a molar ratio of 10:1 and incubated for one hour. The quantity of lipid mix was adjusted at a lipid-to-protein ratio (LPR, in m:m) of 0.5. The DDM and Triton X-100 were removed using dialysis with Bio-beads (BioRad). The protein solution was dialyzed against 50 mM Tris-HCl pH 8.0, 100 mM NaCl, and 5% glycerol during 9 days.

For DnaB, the protein was purified by heparin-agarose affinity chromatography (5 mL HiTrap Heparin HP column from GE Healthcare Life Sciences) equilibrated with 10 mM phosphate pH 7.5, 2 mM βME. The protein was eluted using a 0–100 % gradient of 10 mM phosphate pH 7.5, 1 M NaCl, 2 mM βME. Fractions containing the protein were pooled and loaded onto an anion exchange chromatography (5 mL HiTrap Q HP column from GE Healthcare Life Sciences). The purified protein was concentrated up to 30 mg mL^{-1} by centrifugation in buffer A (2.5 mM sodium phosphate, pH 7.5, 130 mM NaCl).

Preparation of the DnaB+AMPPCP/ DnaB+AMPPNP and BmrA+AMPPCP/ BmrA+AMPPNP

BmrA or DnaB in were mixed with 5 mM $MgCl_2$ and consecutively 15 mM AMPPCP or AMPPNP and incubated for 2 hours at 4 °C.

Preparation of the DnaB+ADP+Vi and BmrA+ADP+Vi

For the preparation of the Protein+ADP+Mg+Vi (NMR samples) or Protein+ADP+Mn+Vi (EPR samples) complexes, the protein were incubated with 1 mM Na_3VO_4, then 10 mM ATP (BmrA) or 10

mM ADP (DnaB) and 10 mM Mg^{2+} or 1 mM Mn^{2+}during 1 hour at 4 ℃. The preparation of vanadate solution was carefully done as described in the literature[134].

Preparation of the DnaB+ADP+AlF_X and BmrA+ADP+AlF_X

DnaB or BmrA was mixed with 5 mM $MgCl_2$ and consecutively 6 mM of an NH_4AlF_4 solution (prepared by incubating 1 M $AlCl_3$ solution with a 5-fold excess of 1 M NH_4F solution (compared to $AlCl_3$) for 5 min. in H_2O) and 5 mM ATP (BmrA) or ADP (DnaB) and incubated for 2 hours at 4 °C.

Preparation of DnaB+nucleotide+DNA

DnaB:nucleotide complexes were prepared as described above 1 mM of (dT)20 was added to the complexes and reacted for 30 min at room temperature.

Solid-state NMR experiments

For solid-state NMR, the protein solutions were sedimented in the MAS-NMR rotor (16 h at 4 °C at 210,000 × *g* for DnaB, 1 h at 4 °C at 210,000 × *g* for BmrA) using home-build rotor filling tools[15]. ^{13}C-detected solid-state NMR spectra were acquired at 20.0 T static magnetic field strength using a 3.2 mm Bruker Biospin E-free probe[135]. ^{31}P-detected and ^{51}V-detected experiments were acquired at 11.7 T in a Bruker 3.2 mm probe. The MAS frequency was set to 17.0 kHz or 19.0 kHz. The 1D and 2D spectra were processed with the software TOPSPIN (version 3.5, Bruker Biospin). DARR spectra were processed with a shifted (3.0) squared cosine apodization function and automated baseline correction in the indirect and direct dimensions. The sample temperature was set to 278 K. All spectra were analysed with the software CcpNmr[136-138] and referenced to 4,4−dimethyl-4-silapentane-1-sulfonic acid (DSS). More experimental details can be found in references [82, 92].

EPR experiments

For EPR experiments, DnaB was concentrated to 48 mg/ml (850 μM) using a Vivaspin 500 centrifugal filter with a cut-off of 30 kDa. Then, the concentrated protein was incubated in presence of ADP 6 mM, Mn^{2+} 170 μM and vanadate 7 mM 2h at 4 ℃. After 2h, glycerol was added to a concentration of 20 %. The final concentration of DnaB was 690 μM, ADP 5 mM, Mn^{2+} 138 μM and vanadate 6 mM.

For BmrA, the protein was not reconstituted in B. subtilis lipids. The protein in 50 mM Tris-HCl pH 8.0, 100 mM NaCl, 10% glycerol (v/v), and 0.2% DDM (m/v) was concentrated to 18 mg/mL (270 μM) using ab Amicon Ultra Centrifugal filter with a cut-off of 50kDa. Then, the concentrated protein is incubated in presence of ATP 900 μM, Mn^{2+} 150 μM and/or Vanadate 400 μM.

2-3 μl of protein or background solution were transferred into an 0.9 mm OD quartz capillary and flash frozen in liquid nitrogen before insertion into the spectrometer. All experiments were conducted on a Bruker Elexsys E680 X-/W-band spectrometer using a EN 680-1021H resonator. The temperature was controlled with a Helium-flow cryostat (ER 4118 CF, Oxford Instruments) and generally set to 10 K. All measurements were conducted at W-band frequencies (~94 GHz, corresponding to ~145 MHz proton Larmor frequency). The shot repetition time was 1 ms.

Echo-detected field-swept (EDFS) EPR spectra were acquired with a Hahn echo sequence, $t_p - \tau - 2t_p - \tau$ – echo, with $t_p = 16$ ns, $\tau = 400$ ns and 200 ns integration window.

Electron-electron double resonance (ELDOR)-detected NMR spectra were acquired with the echo-detected hole-burning sequence $t_{HTA} - T - t_p - \tau - 2t_p - \tau$ – echo, with $t_{HTA} = 50$ μs, $T = 10$ μs, $t_p = 100$ ns, $\tau = 1400$ ns and an integration window of 1400 ns. The frequency of the high-turning angle (HTA) pulse was incremented in steps of 0.1 MHz over the measured range. A +/- phase cycle on the first $\pi/2$ pulse of the echo was used to eliminate unwanted coherence transfer pathways. The power of the HTA pulse, generated by the ELDOR channel of the spectrometer, was optimized such that the observed lines were as intense as possible without broadening them. The nutation frequency ν_1 at the center of the resonator was about 6 MHz. The settings were held constant between protein samples and the corresponding control. Yet it is important to note that exact reproducibility of peak intensities between runs may be difficult with the resonator used because the resonator profile strongly affects line intensities in EDNMR, and hence a careful experimental setup is required.

Raw EDNMR data were background corrected with a Lorentzian line that was fitted to the central hole, and normalized to the signal intensity far off-resonance, i.e. the peak intensity corresponds to the relative hole depth.

Supplementary Figures

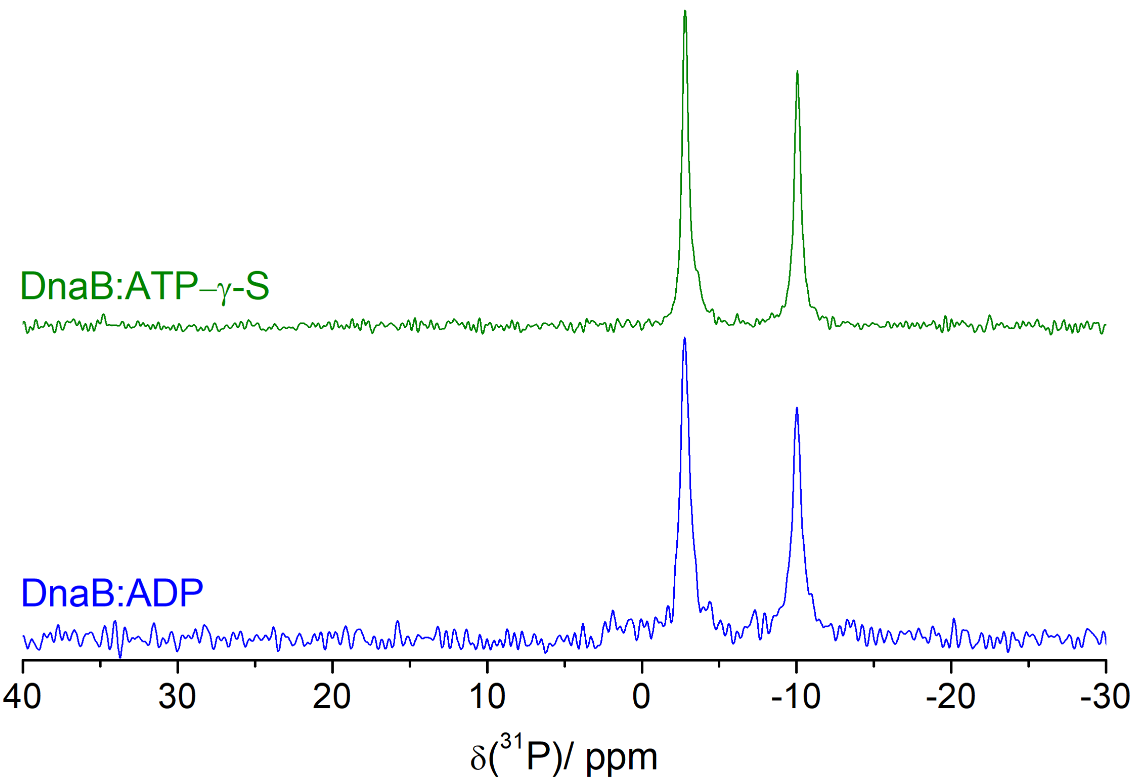

Figure S1: *ATP-γ-S is hydrolysed by DnaB during MAS rotor filling.* ^{31}P,^{1}H CPMAS spectra of DnaB:ADP and DnaB:ATP-γ-S recorded directly after filling the MAS rotor over night. Both spectra are identical indicating the formation of DnaB:ADP in both cases.

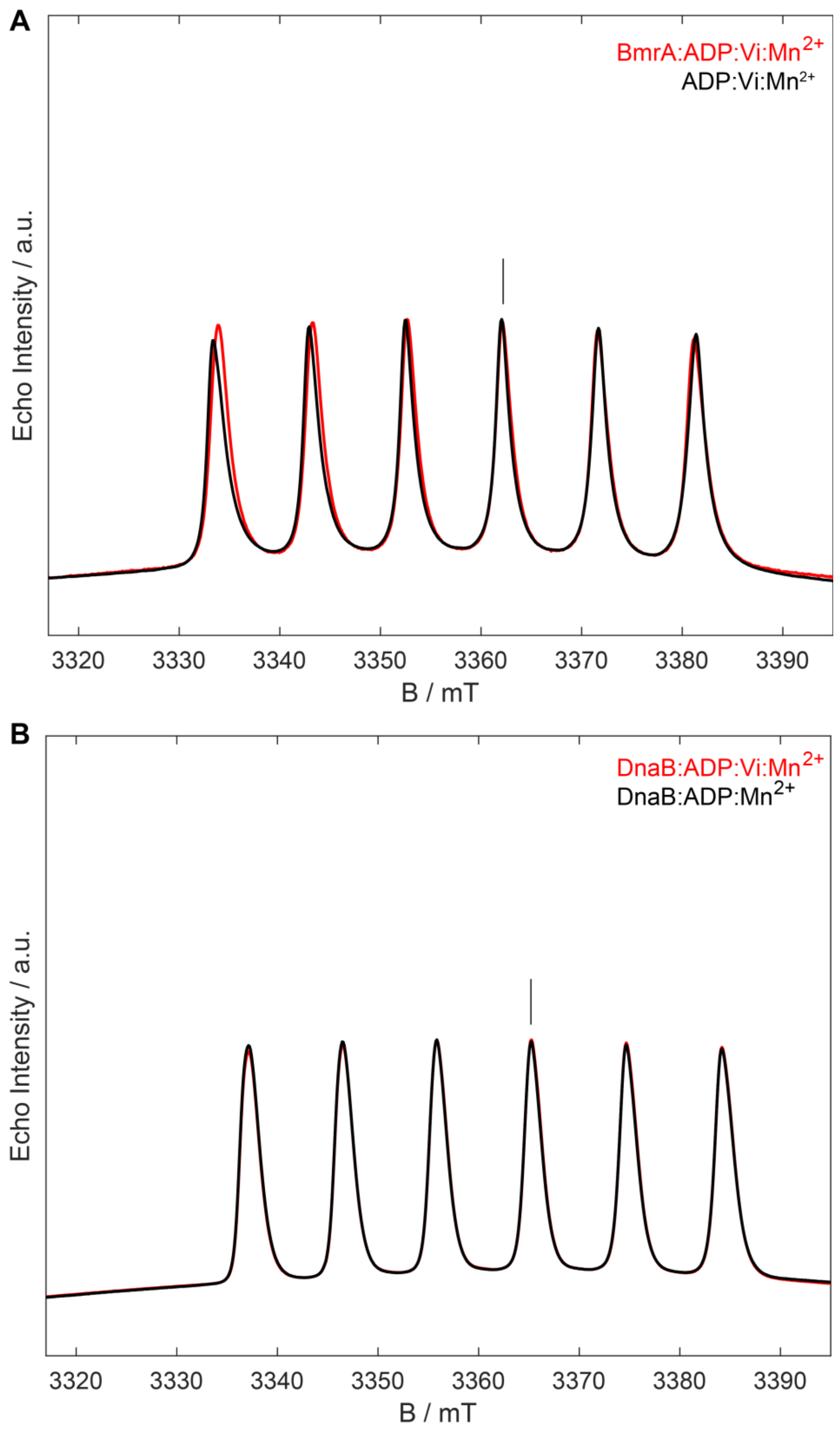

Figure S2: Echo-detected field-swept EPR spectra of BmrA:ADP:Vi:Mn^{2+} and a control solution in the absence of protein (**A**) and of DnaB:ADP:Vi:Mn^{2+} and DnaB:ADP:Mn^{2+} (**B**).

References

1. Lacabanne, D.; Kunert, B.; Gardiennet, C.; Meier, B. H.; Böckmann, A., Sample Preparation for Membrane Protein Structural Studies by Solid-State NMR. *Methods Mol. Biol.* **2017**, *1635*, (7402), 345-358.
2. Gordon, J. A., Use of vanadate as protein-phosphotyrosine phosphatase inhibitor. In Methods in Enzymology, Academic Press: **1991**; Vol. 201, pp 477-482.
3. Gor'kov, P. L.; Witter, R.; Chekmenev, E. Y.; Nozirov, F.; Fu, R.; Brey, W. W., Low-E probe for (19)F-(1)H NMR of dilute biological solids. *J Magn Reson* **2007**, *189*, (2), 182-9.
4. Fogh, R.; Ionides, J.; Ulrich, E.; Boucher, W.; Vranken, W.; Linge, J. P.; Habeck, M.; Rieping, W.; Bhat, T. N.; Westbrook, J.; Henrick, K.; Gilliland, G.; Berman, H.; Thornton, J.; Nilges, M.; Markley, J.; Laue, E., The CCPN project: an interim report on a data model for the NMR community. *Nat Struct Biol* **2002**, *9*, (6), 416-8.
5. Stevens, T. J.; Fogh, R. H.; Boucher, W.; Higman, V. A.; Eisenmenger, F.; Bardiaux, B.; van Rossum, B. J.; Oschkinat, H.; Laue, E. D., A software framework for analysing solid-state MAS NMR data. *J Biomol NMR* **2011**, *51*, (4), 437-47.
6. Vranken, W. F.; Boucher, W.; Stevens, T. J.; Fogh, R. H.; Pajon, A.; Llinas, M.; Ulrich, E. L.; Markley, J. L.; Ionides, J.; Laue, E. D., The CCPN data model for NMR spectroscopy: development of a software pipeline. *Proteins* **2005**, *59*, (4), 687-96.
